# Smooth muscle cell mineralocorticoid receptor does not contribute to atherosclerosis in male or female ApoE-knockout mice

**DOI:** 10.1371/journal.pone.0360618

**Published:** 2026-10-15

**Authors:** Nicole L. Svedberg, Wenxi An, Sorelle Tan, Iris Z. Jaffe

**Affiliations:** 1 Molecular Cardiology Research Institute, Tufts Medical Center, Boston, Massachusetts, United States of America; 2 Graduate School of Biomedical Sciences, Tufts University School of Medicine, Boston, Massachusetts, United States of America; 3 Tufts University, Medford, Massachusetts, United States of America; Emory University School of Medicine, UNITED STATES OF AMERICA

## Abstract

**Background:**

Young women have a lower risk of myocardial infarction (MI) compared to age-matched men, protection that is lost with aging or obesity. There are sex differences in human plaque histopathology, where male plaques are more inflamed and female plaques have more smooth muscle cell (SMC) content. The hormone aldosterone activates the mineralocorticoid receptor (MR) to regulate gene transcription. Elevated aldosterone is seen with obesity and associates with significantly increased incidence of MI. Cell-type specific knockout (KO) of MR reveals a role for endothelial cell and myeloid MR in atherogenesis and plaque inflammation in male, but not in female, mice. SMC-KO of the MR did not impact plaque phenotype in male mice, but female mice have never been studied. Thus, the role of SMC-MR in atherosclerosis was tested for the first time in female mice, directly comparing male and female littermates to identify potential sex differences.

**Methods and results:**

Atheroprone male and female mice with SMC-specific MR deletion were generated by crossing ApoE-KO mice to MRflox/flox×SMA-Cre-ERT2+/− mice to generate ApoE-KO/SMC-MR-KO and ApoE-KO/SMC-MR-intact littermate controls. Significant knockdown of MR mRNA expression was confirmed in aorta from male and female littermates in this model. After 8 weeks high fat diet (HFD), SMC-MR-KO did not impact aldosterone levels, blood pressure, fasting glucose and cholesterol, or body weight, although males weighed significantly more than females. Atherosclerotic plaque size and phenotype were characterized histologically in aortic root sections. SMC-MR-KO did not significantly impact aortic root plaque area in male or female mice, although females had larger plaques than males. Analysis of Oil-Red-O-stained aortic root sections revealed no significant sex or genotype difference in the percentage of the plaque made up of necrotic core or neutral lipids. Analysis of Masson’s Trichrome-stained sections showed no impact of SMC-MR-KO on the percentage of the plaque composed of fibrosis. Finally, immunofluorescent staining of aortic root sections showed no impact of SMC-MR-KO on the percentage of the plaque that was positive for smooth muscle actin or Mac2, neither of which differed significantly by sex.

**Conclusion:**

In ApoE-KO mice fed HFD for 8 weeks, SMC-MR-KO does not impact traditional cardiovascular risk factors or atherosclerotic plaque size or plaque composition in early atherosclerosis in either sex.

## Introduction

Young women have a lower risk of myocardial infarction (MI) compared to age-matched men. This relative protection is lost after menopause and also in young women with obesity and metabolic dysfunction. As a result MI risk is rising in middle aged women, with worse outcomes compared to men [1]. Postmortem studies demonstrate that MI is most commonly caused by rupture of inflamed atherosclerotic plaques. In humans, there are sex differences in plaque burden and in plaque composition, with plaques from males being more diffuse and with greater inflammation and plaques from females having greater maximum size with more smooth muscle cell and extracellular matrix content [2–6], reviewed in [1]. Endothelial cells (ECs) interact with leukocytes to contribute to plaque inflammation and smooth muscle cells (SMCs) migrate into the plaque to contribute to plaque size, fibrosis, calcification and other phenotypes that may be more critical to plaque pathology in women.

Aldosterone is a hormone that regulates renal electrolyte and fluid balance to control blood pressure. Aldosterone levels are elevated in people with obesity, and dysregulated aldosterone is common with aging and contributes to hypertension [7]. Elevated aldosterone associates with significantly increased incidence of MI compared to blood pressure matched individuals with essential hypertension, suggesting a blood pressure-independent role of aldosterone in MI risk [8,9]. Aldosterone acts by binding to intracellular mineralocorticoid receptors (MR) to regulate gene transcription. In addition to the kidney epithelial cells, MR is expressed in cardiomyocytes, ECs, leukocytes, SMCs, and other cells and tissues that contribute to cardiovascular disease. Multiple studies in atherosclerosis models show that MR antagonism decreases atherosclerosis burden [10–12]. To determine the cell types involved in the benefits of MR inhibition, cell-type specific knockout (KO) mice were used to show that MR in endothelial and myeloid cells contributes to atherosclerotic plaque inflammation in male, but not in female, mice [10,13]. SMC-specific KO of the MR did not impact plaque phenotype in male mice [14], but female mice have never been studied. MR is expressed and rises with age in human aortic SMCs from male and female mice and humans [15]. Thus, we investigated the role of the SMC-MR in atherosclerosis in ApoE-KO mice fed high fat diet (HFD) to induce obesity and hyperlipidemia. We examined the impact of SMC-MR deficiency on plaque parameters in female mice, directly comparing to male littermates, to identify potential sex differences.

## Methods

### Mouse models

All mouse studies were approved by the Tufts University Institutional Animal Care and Use Committee by written consent under protocol B2023-96 and were conducted in accordance with the National Institutes of Health Guide for the Care and Use of Laboratory Animals. All mice are on the C57Bl/6J background. Our previously validated MR^flox/flox^×SMA-Cre-ERT2^+/−^ mice [16] were crossed to ApoE-KO mice to generate ApoE-KO/MR^flox/flox^/Cre+ (SMC-MR-KO) and ApoE-KO/MR^flox/flox^/Cre- (SMC-MR-intact) littermate controls as previously described [14]. All male and female Cre- and Cre+ mice were treated with tamoxifen (intraperitoneal injection, 1 mg, daily for 5 days at 6–8 weeks of age) to induce the Cre recombinase in the Cre+ mice and then fed HFD (Envigo Teklad TD.88137, 0.2% cholesterol, 42% fat) for 8 weeks. As ApoE-KO mice do not develop plaques in their coronary arteries, death was not an expected outcome of the study and humane endpoints were not set. Mouse health and behavior were monitored daily by veterinary staff, and no mice died during the course of the study. At the end of the study after 8 weeks of HFD, all mice were euthanized under 4% isoflurane and aortic root tissue frozen in OCT for histological analysis described below.

### Quantitative RT-PCR

Mouse descending aortas were snap frozen and stored at −80° C at time of harvest. Frozen tissue was homogenized in QIAzol lysis reagent (QIAGEN). After chloroform phase-separation, the aqueous phase was transferred to a fresh tube, mixed with 100% ethanol, and total RNA was purified using the RNeasy Mini Kit (QIAGEN) according to the manufacturer’s instructions. RNA was eluted in RNase-free water and quantified using a NanoDrop spectrophotometer. One-step RT-qPCR was performed using iTaq Universal SYBR Green One-Step Kit (Bio-Rad). MR and GAPDH primers are listed below:

MR_forward: AACCACACGGTGACCTGTCATC
MR_reverse: AGTGACACCCAGAAGCCTCATC
GAPDH_forward: CATCACTGCCACCCAGAAGACTG
GAPDH_reverse: ATGCCAGTGAGCTTCCCGTTCAG

### Traditional risk factors

#### Metabolic measurements.

Fasting blood was collected at the end of each study. Fasting glucose was measured by glucometry (TrueBalance). Fasting total cholesterol was measured by Amplex Red Cholesterol Assay Kit (Invitrogen), as previously described [13,17]. Blood was taken at the time of euthanasia by cardiac puncture, and serum aldosterone levels were measured by radioimmunoassay (Tecan, MG13051) as described [18].

#### Physiologic measurements.

Systolic blood pressure was measured by tail cuff plethysmography (CODA System, Kent Scientific) as previously described [10]. We previously validated that this method gives values consistent with telemetry measured blood pressure, and chose tail cuff for blood pressure measurement during atherogenesis studies as the aortic telemetric catheter can impact atherogenesis by altering blood flow characteristics in the proximal aorta [16].

### Aortic root histology and immunofluorescence

Hearts were embedded in OCT and frozen sections at the level of the aortic root were used to analyze plaque histology to ensure that the same region was compared in each mouse. Embedded aortic roots were cut in 10 micron cryosections and serial sections were alternately stained with: Oil-Red-O, to measure plaque size and the percentage of the plaque made up of neutral lipid content, and necrotic core and; Masson’s Trichrome, to measure the percentage of fibrosis; and immunofluorescent staining to measure inflammation (anti-Mac2, Cedarlane, CL8942AF6) and smooth muscle actin content (SMA, Sigma, F3777) in the plaque. Three serial sections, each 30 microns apart were analyzed for each stain, and averaged together for each mouse to represent an N of 1. Plaque size and percent composition were quantified by blinded investigators as we described [10,13,14,17]., Representative photos were chosen based on the image for which the quantification was closest to the average of all images or blots.

### Statistics

All data were collected and analyzed by sex and treatment-blinded investigators. Each *in vivo* study was conducted with at least 3 independent cohorts including all comparator groups in order to support reproducibility. Statistical analysis was performed in GraphPad Prism Version 10. Scatter dot-plots indicate the mean with standard error (SEM) of biological replicates. Data normality and variance were determined by Shapiro-Wilk test. For comparison of four groups, statistical significance was determined by 2-way ANOVA as indicated in each figure legend. Differences were considered statistically significant at p < 0.05.

## Results

### SMC-MR-KO decreases aortic MR expression without impacting aldosterone levels in both sexes

Male and female SMC-MR-intact and SMC-MR-KO littermates were fed HFD for 8 weeks. This duration was chosen based on a previous study demonstrating that 8 weeks of high fat feeding in ApoE-KO mice resulted in aortic root plaque atherosclerosis that could be mitigated by MR-KO in myeloid-cells in both male and female ApoE-KO mice [13]. Knock-out of SMC-MR was confirmed by measurement of MR mRNA in whole aortas by RT-qPCR, showing a significant decrease in MR mRNA in both male and female SMC-MR-KO mice compared to SMC-MR-intact controls (Fig 1A). After 8 weeks of HFD, plasma aldosterone levels and traditional cardiovascular risk factors were measured. There was no significant difference in aldosterone level in males compared to females, nor was the aldosterone level impacted by the presence of SMC-MR (Cre genotype) regardless of sex (Fig 1B).

**Fig 1:**
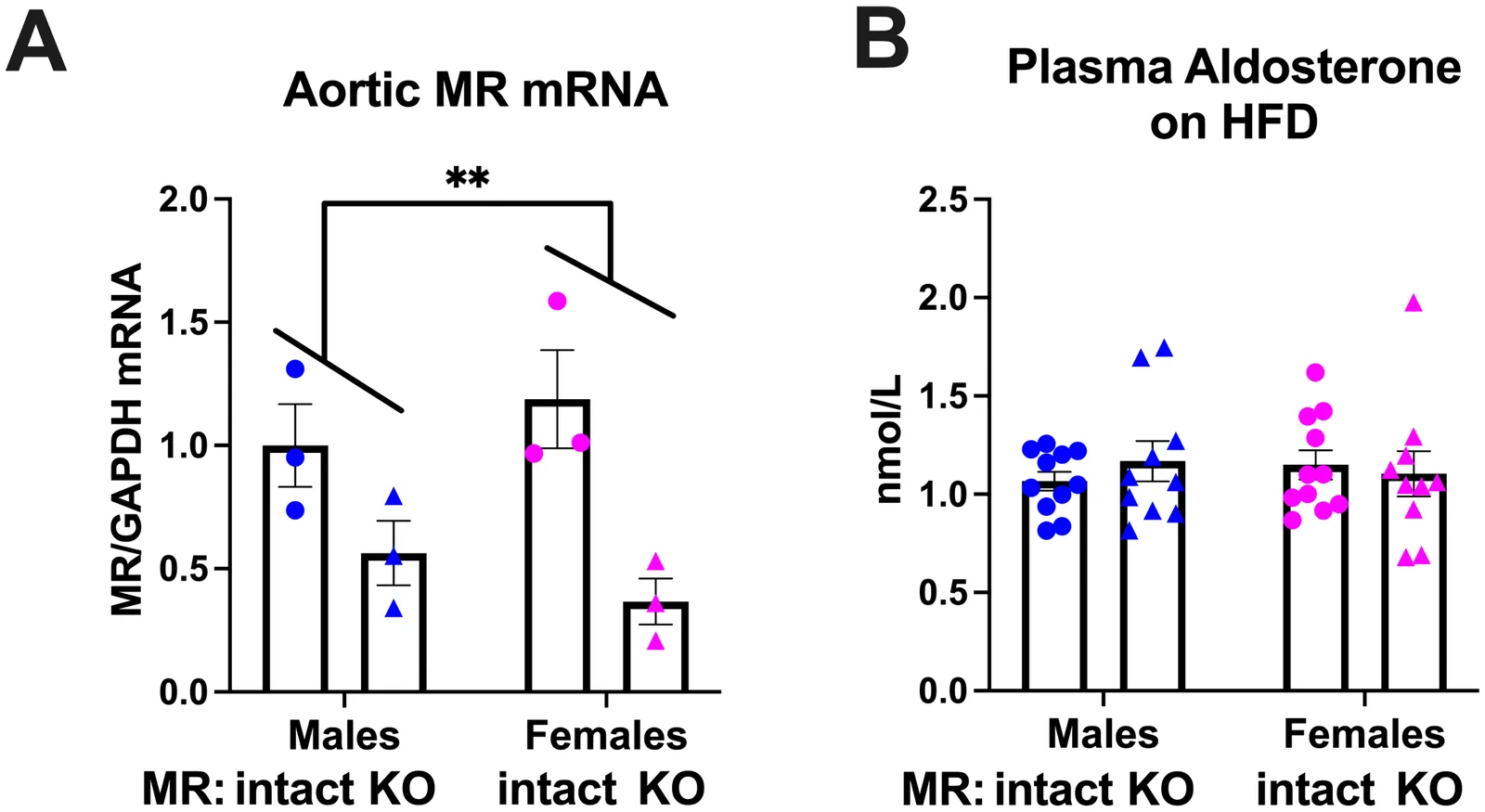
Smooth muscle cell-specific MR knockout (SMC-MR-KO) decreases aortic MR mRNA levels, without impacting serum aldosterone levels. Male and female ApoE-KO/SMC-MR-intact (circles, Cre^−^) and ApoE-KO/SMC-MR-KO (triangles, SMA-Cre-ERT2^+^) littermates were fed with high fat diet for 8 weeks. (A) Aortic MR mRNA was quantified by qRT-PCR and normalized to GAPDH (N = 3). (B) Plasma aldosterone levels (N = 10-11) were quantified and compared in males and females in SMC-MR-intact and SMC-MR-KO mice after 8 weeks of high fat diet. 2-way ANOVA with Bonferroni posttest. P-value <0.05 by genotype is indicated by black bars. ** = p-value < 0.01.

### Smooth muscle cell-specific MR knockout (SMC-MR-KO) does not impact traditional cardiovascular risk factors in males or females

As expected, HFD feeding in ApoE-KO mice induced weight gain, glucose intolerance and severe hyperlipidemia with no significant differences by sex. SMC-MR-KO did not significantly impact the level of fasting cholesterol, fasting glucose, or systolic blood pressure in male and female mice (Fig 2A-C). As expected, male mice weighed more than females, but body weight did not differ by genotype (Fig 2D). Thus, any change in plaque phenotype based on genotype may be attributed to direct effects of MR in SMCs.

**Fig 2:**
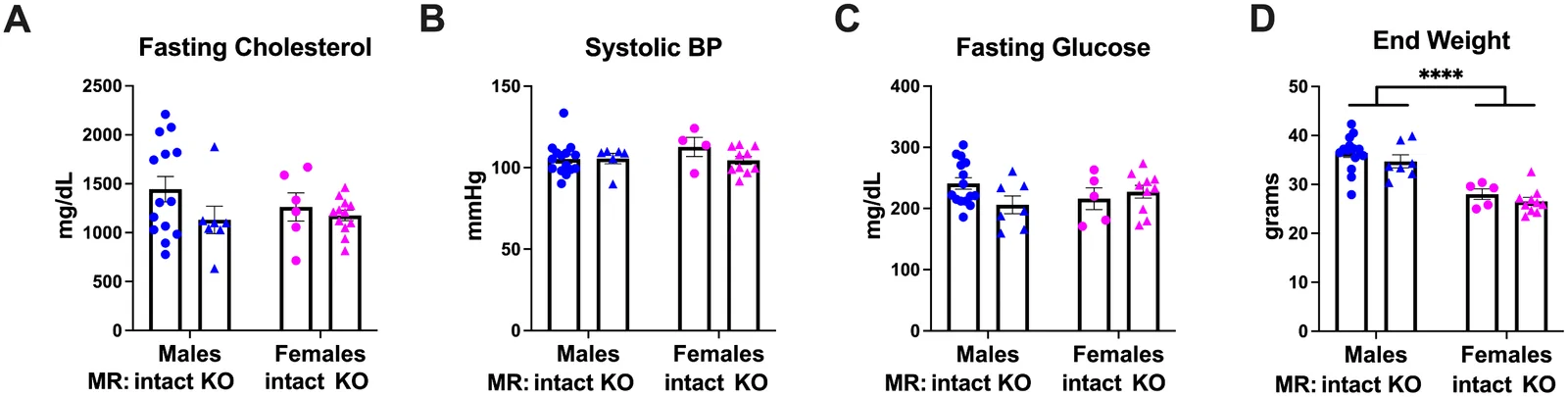
Smooth muscle cell-specific MR knockout (SMC-MR-KO) does not impact traditional cardiovascular risk factors in males or females. Male and female ApoE-KO/SMC-MR intact (circles, Cre^−^) and ApoE-KO/SMC-MR-KO (triangles, SMA-Cre-ERT2^+^) littermates were fed with high fat diet for 8 weeks. Cardiovascular risk factors: fasting cholesterol (A, N = 5-15), systolic blood pressure (B, N = 4-15), fasting glucose (C, N = 5-15) and weight (D, N = 5-15) quantified after 8 weeks of HFD. 2-way ANOVA with Bonferroni posttest. A-C were not statistically significant by sex or genotype. **** = p-value < 0.0001.

### SMC-MR-KO does not impact plaque phenotypes in ApoE-KO mice exposed to HFD for 8 weeks

To assess the impact of SMC-MR-KO on atherosclerotic plaque morphology, aortic root sections were analyzed histologically after 8 weeks of HFD. Females had significantly larger aortic root plaque area compared than males (Fig 3A-B), consistent with prior studies [1]. Further analysis of sections stained with Oil-Red-O quantified the percent of the plaque composed of lipids and necrotic core, markers of plaque instability, revealing no significant difference by sex or genotype (Fig 3C-D). The percent of the plaque made up of collagen-containing fibrosis, a marker of plaque stability, was quantified from sections stained with Masson’s trichrome. There was no significant impact of SMC-MR-KO or sex on the percentage of plaque fibrosis. (Fig 4). Additional frozen sections were stained with antibodies for smooth muscle actin (SMA), a marker of differentiated SMCs which can contribute to fibrous cap stability, and Mac-2 (galectin), a marker of activated macrophages, which contribute to plaque instability. Quantification revealed no significant differences in the percentage of the plaque that stained positive for SMA or Mac-2 staining by sex or genotype (Fig 5). The results reveal that although plaques in female mice are larger, the percentage of the plaque made up of each component, including lipid content, necrotic core, fibrosis, SMA positive and MAC2 positive areas, do not significantly differ by sex, nor do they change when MR is deleted from smooth muscle in either sex.

**Fig 3:**
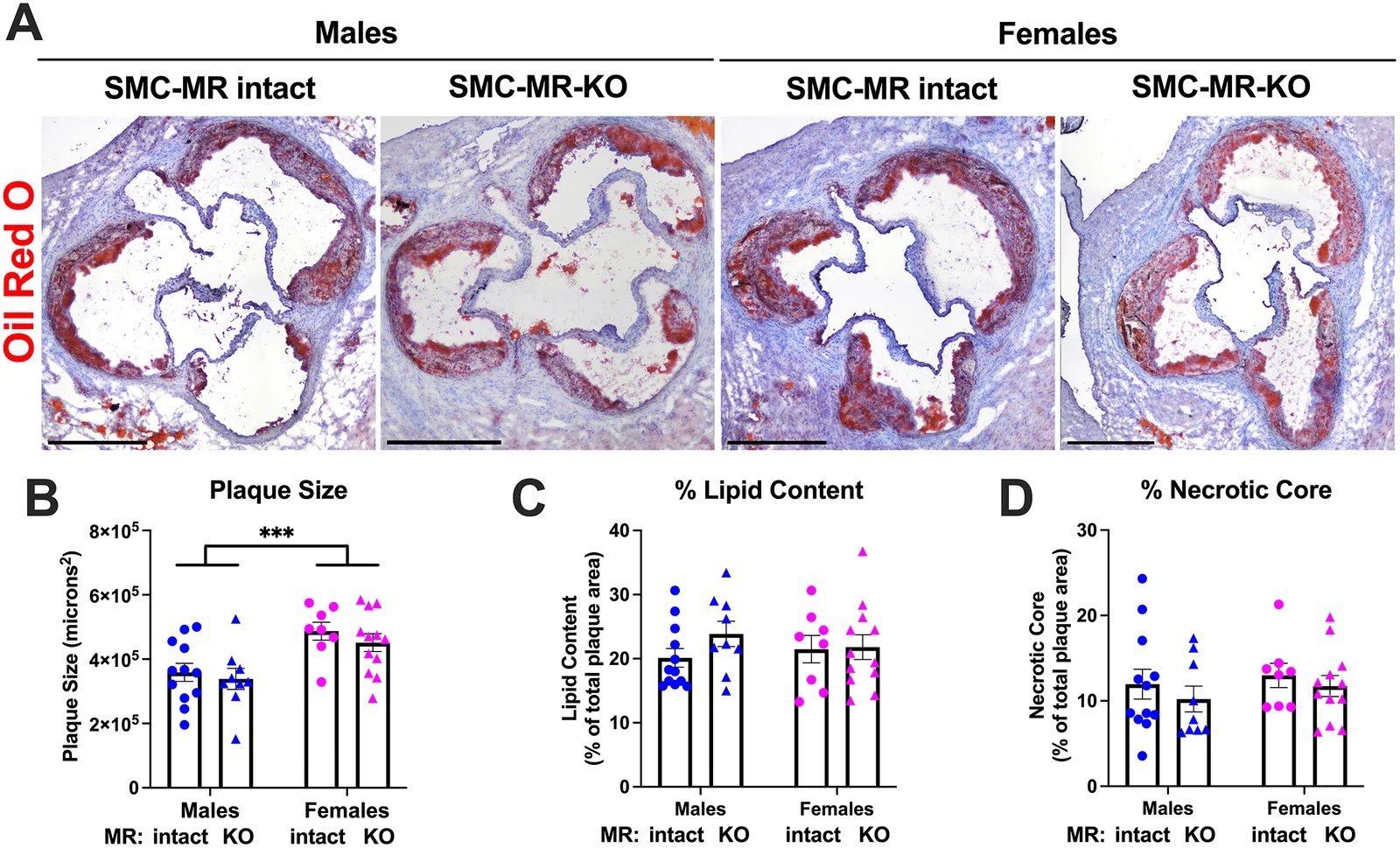
Smooth muscle cell-specific MR knockout (SMC-MR-KO) does not impact plaque size, lipid content, or necrotic core in males or females. Male and female ApoE-KO/SMC-MR intact (circles, Cre^−^) and ApoE-KO/SMC-MR-KO (triangles, SMA-Cre-ERT2^+^) littermates were fed with high fat diet for 8 weeks. Atherosclerotic plaque composition was analyzed in aortic root by histology. (A) Representative Oil-Red-O/H&E-stained stained images with quantification of; (B) plaque cross-sectional area, (C) the percentage of plaque area composed of neutral lipids and (D) of necrotic core (N = 8-12). Scale Bar = 500 microns. 2-way ANOVA with Bonferroni posttest. *** = p-value < 0.0005.

**Fig 4:**
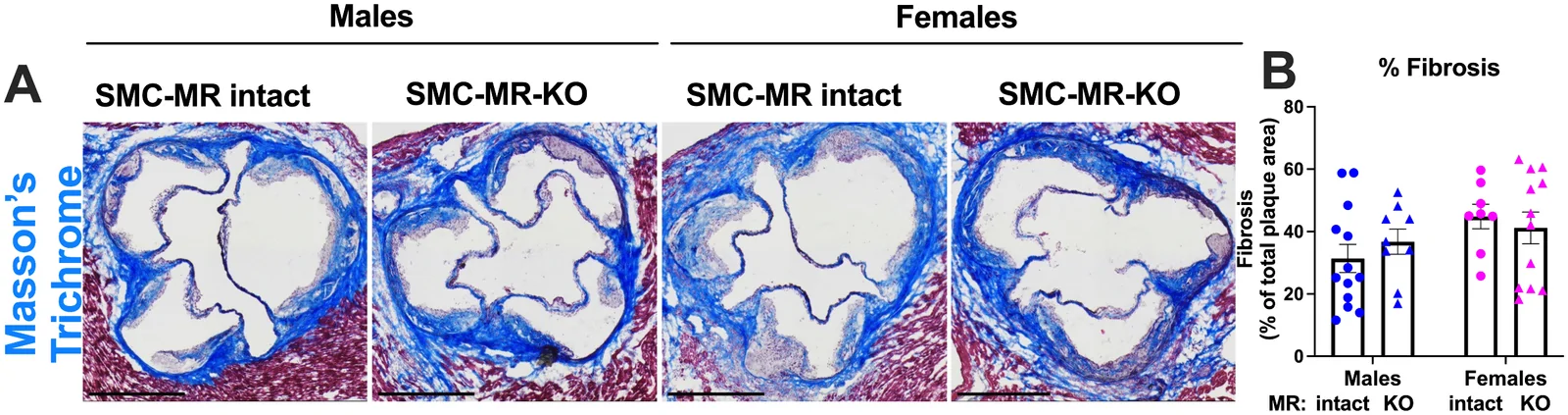
Smooth muscle cell-specific MR knockout (SMC-MR-KO) does not impact fibrosis in males or females. Male and female ApoE-KO/SMC-MR intact (circles, Cre^−^) and ApoE-KO/SMC-MR-KO (triangles, SMA-Cre-ERT2^+^) littermates were fed with high fat diet for 8 weeks. Atherosclerotic plaque composition was analyzed in aortic root by histology. (A) Representative Masson’s Trichrome-stained images of aortic root cryosections. Quantification of (B) the percentage of the plaque made up of fibrosis (N = 8-12). Scale Bar = 500 microns. Not statistically significant by 2-way ANOVA with Bonferroni posttest.

**Fig 5:**
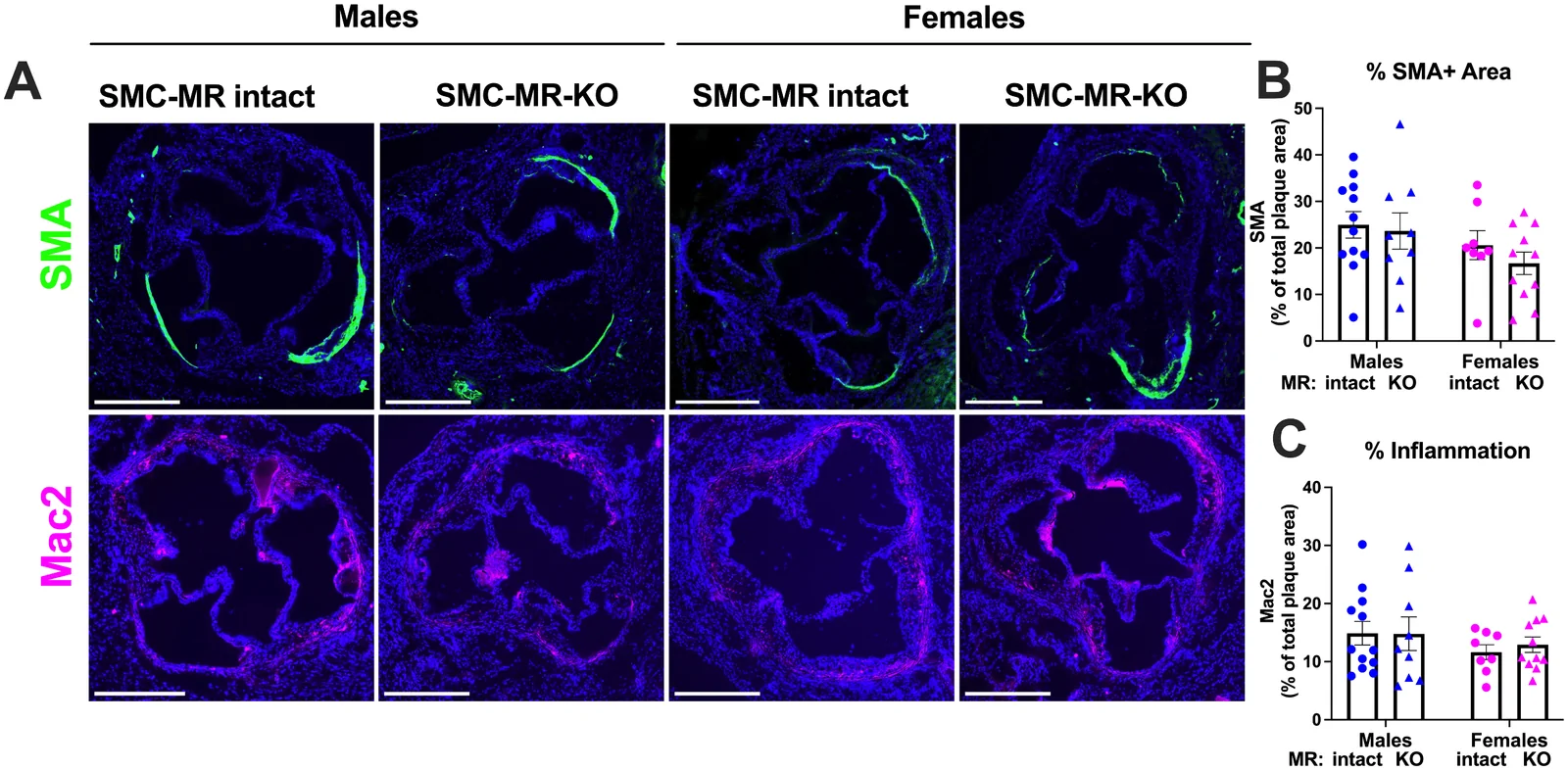
Smooth muscle cell-specific MR knockout (SMC-MR-KO) does not impact plaque SMA or Mac2 content in males or females. Male and female ApoE-KO/SMC-MR intact (circles, Cre^−^) and ApoE-KO/SMC-MR-KO (triangles, SMA-Cre-ERT2^+^) littermates were fed with high fat diet for 8 weeks. Atherosclerotic plaque composition was analyzed in aortic root by immunohistochemistry. (A) Representative smooth muscle actin (SMA) and Mac2 immunofluorescent images of aortic root cryosections. Quantification of the percent of the plaque positive for; (B) SMA+(N = 8-12) and (C) Mac2+ (n = 8-12). Scale Bar = 500 microns. Not statistically significant by 2-way ANOVA with Bonferroni posttest.

## Discussion

This study demonstrates for the first time that SMC-MR does not contribute to atherosclerotic plaque size or phenotype in females in an atherogenic mouse model. By comparing directly to male littermates, the study confirms the lack of impact of SMC-MR in atherogenesis in male mice. Although clinical data correlates MR activation by increased aldosterone with atherosclerotic ischemic events in patients [8,9], that MR inhibition slows progression of plaques in humans [19], and that SMC-driven pathology appears to play a greater role in atherosclerosis phenotypes in women [2,5,6], we find here that SMC-MR does not appear to contribute to plaque histopathology in either sex under these conditions. Aldosterone infusion increases plaque size and inflammation in male mice, although this has never been tested in females [18] while MR inhibition prevents atherogenesis [10–12]. Mechanistically, MR in endothelial and myeloid cells has been found to contribute to plaque inflammation specifically in male mice by upregulating EC adhesion molecules and respective ligands on leukocytes to contribute to leukocyte trafficking into the vasculature, thus driving vascular inflammation [10,13]. Given that EC and myeloid MR, and now SMC-MR do not contribute to atherosclerotic pathology in females, these data support that sex-specific therapies may be needed to prevent unstable plaque phenotypes, with MR playing a greater role in males. However, clinical studies have not separately reported the impact of aldosterone levels or MR inhibition on atherosclerosis phenotypes or outcomes by sex.

There are limitations to that study that should be addressed. This study examined the role of SMC-MR in the early progression of atherosclerosis by measuring outcomes ApoE-KO mice after 8 weeks of HFD. Though this time frame of diet is supported by recommendations for preclinical assessment of atherosclerosis from the American Heart Association [20], it is possible that this time point is too early to assess an impact of SMC-MR on advanced lesion morphology including fibrous cap formation and calcification. Thus, we cannot rule out the possibility that SMC-MR may contribute to atherosclerosis at more advanced disease states. Likewise, though we have replicated previous data in murine models showing that there is not a sex difference in percent of SMA+ plaque area, a study of human carotid endarterectomy specimen has identified increased SMA+ area in females compared with males [2]. Whether this is due to species differences, vascular bed differences, or the study of early atherosclerosis in this model versus later disease in carotid endarterectomy, warrants continued investigation. Additionally, though we measured staining of Mac2 in aortic roots as one readout of inflammation, it is possible that other measurements of plaque stability may be altered even in the absence of changes in Mac2. Lastly, tamoxifen was used to induce SMC-MR recombination in this study, which has been shown to impact atherosclerosis when compared to vehicle control [21]. To address this, all mice, whether Cre- and Cre+, were injected with tamoxifen in this study, thereby mitigating tamoxifen as a potential confounding variable.

Future studies should explore other potential regulators of SMC function in atherogenesis in females, directly comparing to male littermates to identify sex differences in the biology of atherogenesis. Given that our mechanistic understanding of sex differences in cardiovascular ischemic events remains underdeveloped, it is important to continue directly comparing males and females to determine whether mechanistic insights might be sex-specific with the ultimate goal of developing precision strategies to prevent and treat MIs to yield better outcomes in both sexes.

## Abbreviations

ApoE: Apolipoprotein E
EC: endothelial cell
CVD: cardiovascular disease
HFD: high fat diet
KO: knockout
MI: myocardial infarction
MR: mineralocorticoid receptor
SMC: smooth muscle cell.
